# Photophysical, Thermal and Structural Properties of Thiophene and Benzodithiophene-Based Copolymers Synthesized by Direct Arylation Polycondensation Method

**DOI:** 10.3390/polym13071151

**Published:** 2021-04-04

**Authors:** Newayemedhin A. Tegegne, Zelalem Abdissa, Wendimagegn Mammo

**Affiliations:** 1 Department of Physics, Addis Ababa University, Addis Ababa P.O. Box 1176, Ethiopia; 2 Department of Chemistry, Addis Ababa University, Addis Ababa P.O. Box 1176, Ethiopia; zelalem2ab@yahoo.com (Z.A.); wendimagegn.mammo@aau.edu.et (W.M.)

**Keywords:** intramolecular charge transfer, copolymers, *π* − *π* stacking, direct arylation polycondensation, and excitonic state

## Abstract

Three low-band-gap copolymers based on isoindigo acceptor units were designed and successfully synthesized by direct arylation polycondensation method. Two of them were benzodithiophene (BDT)-isoindigo copolymers (**PBDTI-OD** and **PBDTI-DT**) with 2-octlydodecyl (OD) and 2-decyltetradecyl (DT) substituted isoindigo units, respectively. Thiophene donor and DT-substituted isoindigo acceptor units were copolymerized to synthesize **PTI-DT**. The copolymers have a broad absorption range that extends to over 760 nm with a band gap ≈1.5 eV. The photophysical property studies showed that the BDT-based copolymers have non-polar ground states. Their emission exhibited the population of the intramolecular charge transfer (ICT) state in polar solvents and tightly bound excitonic state in non-polar solvents due to self-aggregation. On the contrary, the emission from the thiophene-based copolymers was only from the tightly bound excitonic state. The thermal decomposition temperature of the copolymers was above 380 °C. The X-ray diffraction pattern of the three copolymers showed a halo due to π−π stacking. A second, sharper peak was observed in the BDT-based copolymer with a longer side chain on the isoindigo unit (**PBDTI-DT**), and the thiophene-based copolymers with **PTI-DT**, exhibiting a better structural order.

## 1. Introduction

The introduction of π-conjuageted polymers to the fast-growing solar cell technology has brought forth new features of flexibility and solution processabiltiy in the so-called organic solar cells (OSCs) devices. OSCs have shown a leap in efficiency in the last five years to above 18% in single-junction [1], and over 17% in multi-junction [2], devices. This tremendous improvement is mainly attributed to the introduction of new donor polymers and non-fullerene acceptors that can harvest the broad solar spectrum with appropriate energetics in the active layer. A vast number of polymers have been synthesized and characterized to enrich the structure-property relation recipe for efficient OSCs. Alternating donor–acceptor (D–A) copolymers are commonly synthesized to obtain low-band-gap polymers with good solar harvest that extends to the near-infra-red region. Some important parameters that need to be addressed during the synthesis of the D–A copolymers include the HOMO level, which should be lower-lying, with the LUMO lying in the region for efficient exciton dissociation.

The synthesis of low-band-gap polymers using a simple and economically viable technique has gained the attention of researchers due to its potential for industrialization of the OSC technology. The direct arylation polycondensation method (DAP) has notably shortened the synthesis route compared to the commonly used Suzuki and Stille cross-coupling techniques. In this reaction, the unsubstituted arylene monomers are directly coupled with dihaloarylene units with the help of transition metals—usually palladium acetate. The by-product at each of the synthesis steps is, therefore, drastically reduced, making the DAP method more environmentally friendly and economically viable. In addition, the DAP method made the synthesis of polymers that were difficult to prepare with the traditional Suzuki and Stille cross-coupling techniques possible [3]. The mass production of OSCs will need an enormous amount of donor and acceptor organic materials. An easy and fast synthesis method such as DAP is undoubtedly important in the realization of the commercialization of OSC technology. Despite its many interesting merits, the method has a drawback: its selectivity between C–H bonds for some monomers is poor. Consequently, the method is more commonly used to synthesize homopolymers like poly-3-hexylthiophene (P3HT) and benzodithiophene (BDT) [4,5,6]. However, D–A copolymers synthesized by DAP method are limited. Recently, our group and others have reported the successful synthesis of D–A copolymers using the DAP method [7,8,9,10].

The choice of D and A units in the synthesis of D–A copolymers determines their electrical, optical and structural properties, which that will subsequently determine the performance of the OSC that the materials are used for. Isoindigo is a widely used acceptor unit due to its good electron-withdrawing properties. The fused ring benzo[1,2-b:4,5-b${}^{\prime }$]dithiophene (BDTs) and thiophene units are the most commonly used donor units in the synthesis of D–A copolymers that produced high power conversion efficeny (PCE) of over 11% in OSCs [11]. These polymers are usually contain aloxy or alkyl side chains to enhance their solubility. A basic understanding of the structure–property relationships of the polymers is vital for the optimization of their structure for higher PCE of OSCs. The optical properties, intra/inter-chain interactions in the polymers, and their morphologies in the films are some of the parameters that play important roles in the photogeneration of charges in OSCs. In addition, their thermal properties determine their lifetimes in harsh environment conditions, and also their application in flexible OSC devices, which are normally processed at temperatures below 150 °C.

In this work, we report the synthesis of three D–A copolymers based on an isoindigo acceptor unit. The two copolymers have alkyl-substituted BDT (**PBDTI-OD** and **PBDTI-DT**) donor units, while in the third copolymer (**PTI-DT**), an alkyl-substituted thiophene unit is used. The structures of the BDT-based copolymers were systematically tailored by increasing the side chain length in the isoindigo units from 2-octyldodecyl (OD) in **PBDTI-OD** to 2-decyltetradecyl (DT) in **PBDTI-DT**, while keeping the backbone structure the same. The third copolymer, **PTI-DT**, was synthesized to study the effect of electron-donating groups by changing the donor units from BDT to thiophene. The structural differences in the three copolymers allowed us to investigate the effect of backbone structure on their photo-physics, thermal and structural properties. We found two relaxation channels in the BDT-based copolymers, while a one-channel relaxation was found in **PTI-DT**. The copolymers showed excellent thermal stability with decomposition temperatures (T${}^{D}$ = 5% mass loss) above 380 °C. The X-ray diffraction patterns of the copolymers suggested that a better structural ordering was found in the thiophene-based copolymer.

## 2. Materials and Methods

### 2.1. Materials and Synthesis

**Scheme 1:**
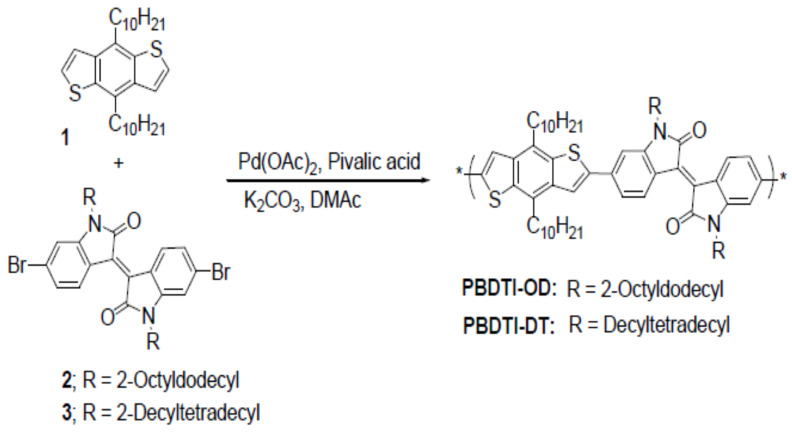
Syntheses scheme of **PBDTI-OD** and **PBDTI-DT**.

Three copolymers based on an isoindigo acceptor were designed and synthesized using a direct heteroarylation polymerization (DAP) method for the first time. The two copolymers were synthesized by the copolymerization of a 4,8-bis(decyl)benzo[1,2-b;4,5-b${}^{\prime }$]dithiophene (BDT) (**1**) donor unit and an isoindigo acceptor unit. N-Alkylation of the isoindigo moiety was achieved with 2-octyldecylbromide and 2-decyltetradecylbromide to obtain compounds **2** and **3** which were used to synthesize **PBDTI-OD** and **PBDTI-DT**, respectively, as shown in Scheme 1. In the synthesis of **PBDTI-OD**/**PBDTI-D**, a 1:1 ratio of BDT (**1**) (0.5 mmol) and isoindigo (**2/3**) (0.5 mmol) was copolymerized using palladium acetate, (0.01 mmol), pivalic acid (0.15 mmol) and potassium carbonate (1.3 mmol) as the catalyst, acidic additive and base, respectively. The polymerization reaction took place in an inert atmosphere at a temperature of 100 °C using anhydrous dimethylacetamide (DMAc) for **PBDTI-DT** and a mixture of DMAc and toluene as the reaction solvents for **PBDTI-OD**. The heating was stopped after 7 and 19 h for **PBDTI-OD** and **PBDTI-DT**, respectively, and the solutions were left to cool down to room temperature. The copolymers were precipitated in methanol and filtered. The crude copolymers were dissolved in chloroform (CF) and washed with aqueous solution of ethylene-diamine tatraacetic acid (EDTA) (pH = 8) to remove trace metals, followed by 0.1 N HCl and distilled water. **PBDTI-OD** was Soxhlet-extracted with methanol, hexane, diethylether and chloroform while **PBDTI-DT** was extracted with methanol, acetone, ethylacetate, hexane, diethylether and chloroform. The CF extract of **PBDTI-OD** was concentrated and precipitated in acetone and filtered. After drying the polymer at 40 °C in a vacuum oven for 24 h, the solid obtained was further stirred in hexane overnight and filtered to obtain 0.36 g blue-black copolymer (56%). Similarly, the CF extract of **PBDTI-DT** was concentrated precipitated in methanol, filtered, dried in an oven at 40 °C for 24 h to afford 0.32 g dark brown solid (43%).

**Scheme 2:**
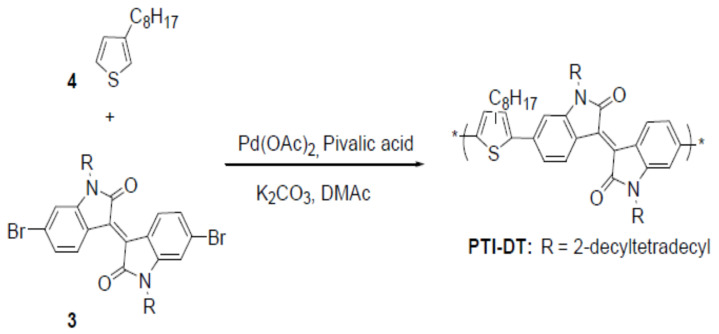
Synthesis scheme of **PTI-DT**.

**PTI-DT** was synthesized by copolymerizing 3-octylthiophene (**4**, 0.5 mmol) with isoindigo (**3**), as shown in Scheme 2. The polymerization was done in anhydrous DMAc for 3.5 h. Following the same procedure, the crude polymer was extracted. **PTI-DT** was Soxhlet-extracted in methanol, acetone, ethyl acetate and CF, which afforded 0.37 g (66%) dark-brown solid after drying in an oven at 40 °C overnight. Note here that the long side chains attached to both the donor and acceptor units of the copolymers increased their solubilities in chlorinated solvents like CF and 1,2-dichlorobenzene (oDCB). Comparing the three copolymers, **PBDTI-OD** was found to be less soluble in these chlorinated solvents like CF and oDCB. On the other hand, introduction of the long side chains might create steric hindrance that might twist the backbones of the copolymers. However, BDT has a planar structure that is expected to make the backbones of **PBDTI-OD** and **PBDTI-DT** less twisted and have a longer conjugation length than the thiophene-based copolymer, **PTI-DT** [12,13].

The molecular weights of the copolymers weree determined by size exclusion chromatography was done using a Waters Alliance GPVC2000 instrument with a refractive index detector. The columns used were Waters Stragel^®^ HMW 6EX2 and Waters Stragel^®^ HT 6EX1 and the measurements were taken in 1,2 4-trichlorobenzene at a temperature of 135 °C. The relative molecular mass was measured against polystyrene standards. The average molecular weights (M${}_{w}$) of **PBDTI-OD**, **PBDTI-DT** and **PTI-DT** were found to be 53,461, 71,234 and 23,574 g/mol, respectively. The high M${}_{w}$s of the copolymers are due to the long alkyl side chains appended both in the donor and acceptor units. The calculated poly-dispersity indices (PDI = M${}_{w}$/M${}_{n}$) of the three copolymers show that **PTI-DT** has a more uniform molecular weight distribution. The molecular weight and yield data are summarized in Table 1.

### 2.2. Absorption and Photoluminescence Spectroscopy

The absorption spectra of **PBDTI-OD**, **PBDTI-DT** and **PTI-DT** in both solutions and spin-coated films were recorded using a Perkin Elmer Lambda 19, UV/Vis/NIR spectrophotometer. The thin films were spin-coated from a CF solution of concentration 10 mg/mL at a spin speed of 1000 rpm and the films were annealed at 85 °C for 10 min. Similarity, their photoluminescence (PL) spectra were recorded both in solutions and spin-coated films using JY Horiba, ‘Fluoromax’-4 spectrofluorometer. The absorption and PL spectra in solutions were taken in CF, o-DCB, and cyclohexane (Chex) with polarity indices of 4.1, 2.7 and 0.2, respectively. The PL spectra of the copolymers in solutions and films were taken at excitations matching their longer wavelength electronic transition. The solutions for PL measurement absorb only 0.05 at the maxima to avoid self absorption.

### 2.3. X-ray Diffraction

The X-ray diffraction (XRD) patterns of the copolymers were recorded for for 2θ values from 2 to 40° in drop-casted films on a glass substrates using Shimadzu Scientific Instruments X-ray diffractometer 7000S with a copper target, Cu-Kα-radiation with 40 kV × 40 mA power. The interlayer spacing between the planes (*d*) was calculated using Bragg’s Equation (1).

(1) nλ=2dsin(θ),

where *n*, λ and θ are order of diffraction (1), wavelength of the X-ray (0.154 nm) and angle of diffraction, respectively.

### 2.4. Thermogravimetric Analysis

The thermal properties of the copolymers were investigated using DTG-60H thermogravimetric analyser in platinum crucibles under nitrogen atmosphere flowing at a rate of 50 mL/min.

### 2.5. Electrochemistry

Square wave voltammetry (SWV) was done to measure the frontier molecular orbitals of the copolyers in a three-electrode setup. Platinium electrodes were used both as working and counter electrodes, while Ag/AgCl was used as a reference electrode, which was calibrated against ferocene/ferrocynium ion (Fc/Fc${}^{+}$). The redox potential of Fc/Fc${}^{+}$ was found to be 0.44 eV versus the reference electrode, taking its energy level to be 4.8 eV below the vacumm. The copolymer films were drop-casted on the Pt disc working electrode, and a 0.1 M solution of tetrabutylammonium hexafluorophosphate ($Bu_{4}NPF_{6}$) in anyhdrous acetonitrile served as a supporting electrolyte. The higherst occupied molecular orbital (HOMO) and lowest unoccupied molecular orbital (LUMO) energy levels were determined from the onsets of oxidation and reduction potentials, respectively, using Equation (2) [14].

(2) $$E_{HOMO/LUMO}=e\left(E_{ox/red}+4.4\right)eV$$

## 3. Results and Discussion

### 3.1. Optical and Electrochemical Properties of PBDTI-OD, PBDTI-DT and PTI-DT

**Figure 1:**
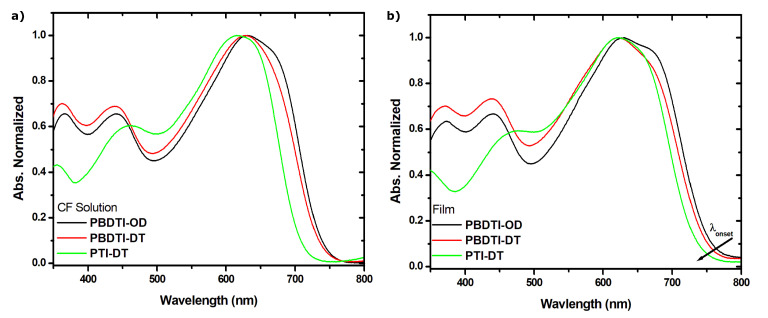
Absorption spectra of **PBDTI-OD** (black), **PBDTI-DT** (red) and **PTI-DT** (green) in (**a**) CF solutions and (**b**) thin films.

The absorption spectra of **PBDTI-OD**, **PBDTI-DT** and **PTI-DT** were taken both in CF solutions and as thin films, as shown in Figure 1a,b. The spectra of **PBDTI-OD** and **PBDTI-DT** in CF solutions are characterized by one dominant transition peaking at 631 and 628 nm, respectively, and two peaks below 450 nm. On the other hand, the absorption spectrum of **PTI-DT** is quite different from the BDT-based copolymers where a dominant one transition in solution that peaks at 615 nm and a modest peak at 450 nm are observed. The two-energy-band profile exhibited in **PBDTI-OD**, **PBDTI-DT** and **PTI-DT** is common in push–pull copolymers. The low energy band is due to the $S_{0}\to S_{1}$ transition, usually with intra-molecular charge transfer (ICT) characteristics due to the charge transfer between the donor and acceptor moieties in the copolymers. On the other hand, the high energy band is due to the $\pi -\pi^{*}$ transition, which is common in the π-conjugated polymers due to the alternating single and double bonds in their backbone [15]. The presumably assigned ICT state transition in **PBDTI-OD** and **PBDTI-DT** is red-shifted by more than 11 nm with respect to **PTI-DT**, suggesting a better electron-donating property of the BDT moiety [16]. This could also be due to the long alkyl side chains on the thiophene and isoindigo units of **PTI-DT** that can sterically intract, leading to a twisting in the backbone of the copolymer. Computaion analusis of a similar BDT-based copolymer showed that the copolymers are likely to adopt a planar structure [16] which facilitates ICT in **PBDTI-OD** and **PBDTI-DT** chains.

The absorption spectra of thin films of **PBDTI-OD**, **PBDTI-DT** and **PTI-DT** were found to be red-shifted by 30, 25 and 40 nm from their solution spectra, respectively. The red-shift in absorbance upon solidification shows a better interchain interaction in the films due to π−π stacking, which is beneficial for charge transport when the copolymers are used in OSCs. The larger red-shift in absorbance of **PTI-DT** upon solidification indicates that a different intra- and intermolecular interaction from the DBT-based copolymers exists due to the thiophene donor unit. One of the reasons for this could be that **PTI-DT** is twisted in the absence of strong intermolecular interaction in dilute solution. However, the stronger inter-molecular force in the solid films pushes the copolymer into a planar conformation, resulting in a siginicant red-shift in absorption onset [17,18]. Similarly, the smaller absorption onset shift in the films of **PBDTI-DT** compared to **PBDTI-OD** shows the more planar structure of **PBDTI-OD** due to the shorter side chain on the isoindigo unit [19]. The optical band gaps (E${}_{g}$) of the three copolymers were calculated from the onsets of absorption spectra of the films and were found to be 1.53, 1.54 and 1.56 eV for **PBDTI-OD**, **PBDTI-DT**, and **PTI-DT**, respectively. The copolymers have a similar bandgap, that is suitable to harvest substantial solar irradiation in the high solar flux region [20]. The backbone structures of the copolymers have negligible effect on their band gaps. The optical properties of the copolymers are summarized in Table 2.

**Figure 2:**
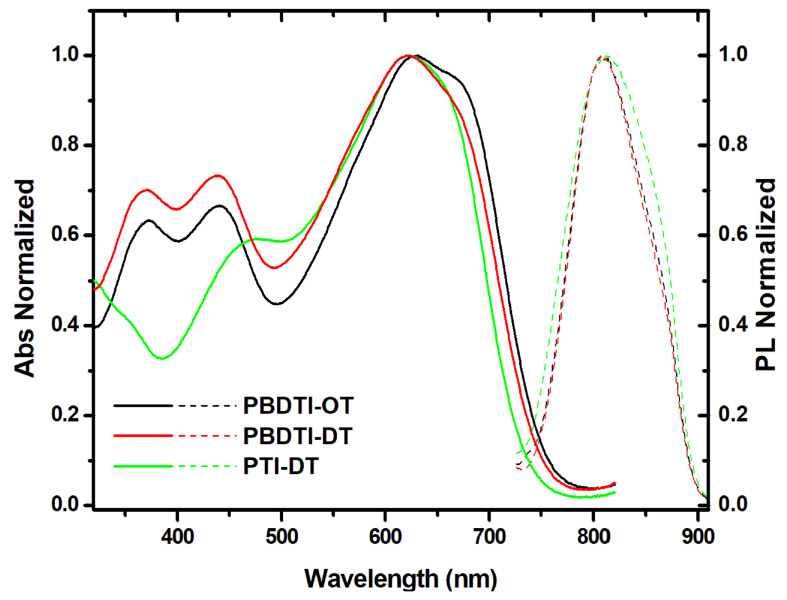
Absorption (solid line) and PL (broken line) spectra of **PBDTI-OD** (black), **PBDTI-DT** (red) and **PTI-DT** (green).

The PL spectra of the thin films were recorded by exciting at their absorption maxima in the longer wavelength region and were found to be Stokes-shifted by 179, 189 and 185 nm in **PBDTI-OD**, **PBDTI-DT** and **PTI-DT**, respectively (see Figure 2). Since an efficeint overalp between the absorption and emission spectra of the copolymers is needed for Förster resonance energy transfer (FRET), the large Stokes shift in the copolymers will inhibit interchain excitation energy transfer [21]. However, the large Stokes shift also shows that multiple processes have taken place to stabilize the first excited state. Since ICT formation in solid films is not efficient, the emission from the first excited state ($S_{0}\leftarrow S_{1}$) is ascribed to tightly bound intrachain exciton relaxation. The strong π−π interaction upon solidfication of the copolymers enables an efficient interchain charge transfer interaction that dominates over intrachain charge transfer (i.e., ICT population). Our work on three similar copolymers with two and three thiophene donor units, coupled with an isoindgo acceptor unit, showed similar results [7,8].

**Figure 3:**
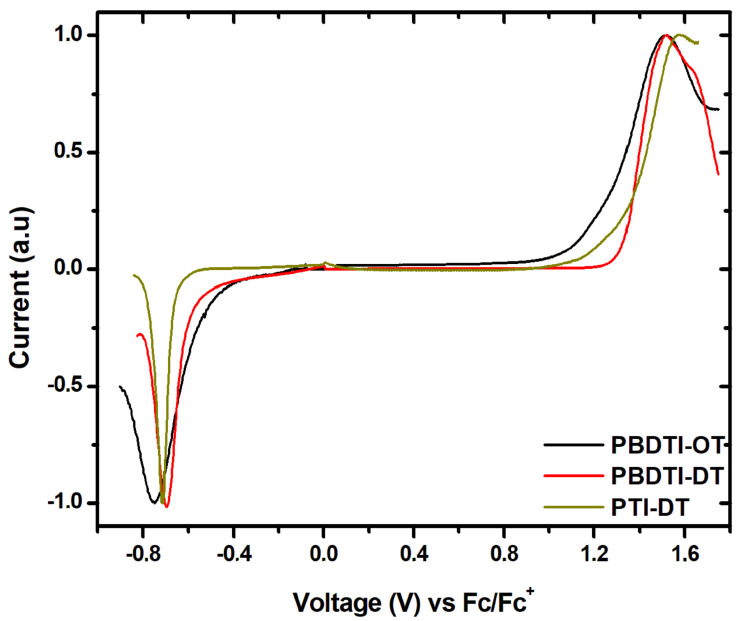
Square wave voltammograms of **PBDTI-OD** (black), **PBDTI-DT** (red) and **PTI-DT** (dark yellow).

The HOMO and LUMO energy levels of the copolymers were determined using SWV from the onsets of their oxidation and reduction potentials, as shown in Figure 3. The HOMO and LUMO energy levels of the copolymers are summarized in Table 2. The difference in the HOMO levels between the copolymers with the same donor unit (**PBDTI-OD** and **PBDTI-DT**) was 0.2 eV, whereas the difference is only 0.1 eV between **PBDTI-DT** and **PTI-DT** (with the same acceptor unit). The highest LUMO level was found in **PBDTI-OD**, followed by **PBDTI-OD** and **PTI-DT**. The π-electron density in the copolymers is not expected to be significantly affected by the alkyl side chains [22]. The reason for the difference in HOMO levels between **PBDTI-OD** and **PBDTI-DT** should, therefore, be due to the effect of the length of the alkyl side chains on the reorganization of the copolymers in the drop-casted films [8,19]. This is expected due to the relatively lower solubility of **PBDTI-OD**.

The difference in the energetics of **PBDTI-DT** and **PTI-DT** is significantly dependent on the electron-donating strength of BDT vesus thiophene units, respectively. The stronger electron-donating property of BDT would enhance the ICT in **PBDTI-DT**, which will stabilize its LUMO level compared to **PTI-DT**, in which the donor unit is a thiophene [23]. Note here that the reorganization of the copolymers in the casted films also plays a role. The medium-lying HOMO levels of the copolymers are beneficial for attaining high open-circuit voltage when they are used in OSCs as donor materials, since $V_{OC}=e\left(E_{LUMO}^{Acceptor}-E_{HOMO}^{Donor}\right)$, where *e* is the elementary charge [24]. The LUMO offset of the copolymers with a fullerene acceptor should be around 0.3 eV for efficient exciton disscociation at the donor/acceptor interface [24,25]. **PBDTI-OD**, **PBDTI-DT** and **PTI-DT** have LUMO offsets of 0.1, 0.2 and 0.3 eV, respectively with respect to the commonly used fullerene acceptor, PC${}_{71}$BM (LUMO = 4.0 eV). The LUMO offsets in the BDT-based copolymers are below the recommended value, which is detrimental to exciton dissociation in fullerene-based OSCs, while efficient exciton dissociation is expected in **PTI-DT**-based OSCs. Hence, **PBDTI-OD** and **PBDTI-DT** might work better when blended with non-fullerene acceptors.

### 3.2. Photophysical Properties of the Copolymers

**Figure 4:**
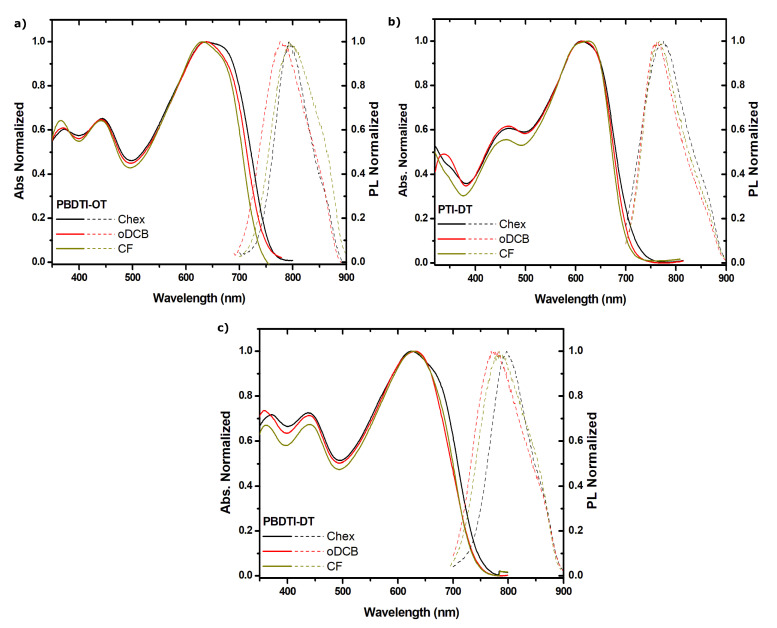
Absorption (solid line) and PL (broken line) of (**a**) **PBDTI-OT**, (**b**) **PBDTI-DT** and (**c**) **PTI-DT** in Chex (black), oDCB (red) and CF (dark yellow) solutions.

To further understand the photophysics of the copolymers, their absorption and PL spectra were recorded in CF, oDCB and Chex solution in a concentration range of 125–5.7 µg/mL. The absorbances of all the copolymers in the three solvents showed negligible shift with concentration gradient, which indicates the absence of aggregation in the solutions. The absorption spectra of the copolymers in the three solvents of different polarity are almost similar, with a slight difference in the apperance of a shoulder when the poor solvent (Chex) was used. However, the low-energy absorption peaks of the copolymers remained the same with increasing solvent polarity, as shown in Figure 4a–c (solid line). This confirms that the ground states (S${}_{0}$) of the copolymers are non-polar. The PL spectra of **PBDTI-OD** showed a red-shift of 18 nm as the solvent polarity increased from 2.7 (oDCB) to 4.2 (CF). The Stokes shift of **PBDTI-OD** in Chex is higher than both oDCB and CF, despite its low polarity index of 0.2. Similarly, a 29 nm red-shift in PL of **PBDTI-DT** is found with increasing solvent polairty from 2.7 (oDCB) to 4.2 (CF). An ever larger Stokes shift in was observed when Chex was used as a solvent. The PL spectra of **PTI-DT** showed negligible shifts in the three solvents.

The red-shift in PL of **PBDTI-OD** and **PBDTI-DT** with increasing solvent polarity while their absorption remained the same confirms the higher dipole moments of their first excited states (S${}_{1}$). This is consistent with our assumption that the longer wavelength region transitions have ICT characterers. Hence, their electronic structure changes from the non-polar D−A to the dipolar $D^{+}=A-$ configuration [15]. The larger Stokes shift observed in Chex solutions of **PBDTI-OD** and **PBDTI-DT** shows the population of a different state than ICT. Consequently, the electrons in the first excited state of the copolymers have two channels; one is the ICT state and the other is the tightly bound excitonic state. The latter is populated when there is a significant interchain interaction either in films or solutions. A poor solvent like Chex is expected to form aggregates that induce interchain interaction in the solutions, resulting in red-shifted PL. Thus, the emission in the non-polar solvent could be explained by the tightly bound interchain exciton model instead of the commonly used ICT model [26,27]. However, the Uv-Vis absorbances of **PBDTI-OD** and **PBDTI-DT** taken in solutions of similar concentration, as the PL measurements confirmed the absence of aggregation. Therefore, the aggregation effect observed in the Chex solutions should be a self-aggregation, causing the copolymer chains to form tight coils. As a result, the excited electrons could be transferred to the other part of the chain acting as interchain excitons [7,28]. Consequently, the PL spectra were red-shifted despite the low polarity of the solvent.

The photophysics of **PTI-DT** is quite different in that both its ground and exited states are not influenced by the polarities of the solvents. The similar Stokes shift, both in polar (CF and oDCB) and the non-polar (Chex) solvents, shows that self-aggregation of the copolymer chain exists in all solvents. **PTI-DT** showed a large absorption onset shift as well as a peak shift, while the BDT-based copolymers showed no peak shift upon solidification (see Table 2). This can be attributed to the twisting of the thiophene-based copolymer in dilute solution in the absence of a strong intermolecular interaction. The twisting in the backbone of **PTI-DT** favours the self-aggregation effect in any solvent. The ICT population from the first excited state of **PTI-DT** is dominated by the excitonic state, making the emitting state insensitive to polarity of the environment.

In summary, the BDT-based copolymers have a bi-relaxation channel that can be modulated with the solvents, while **PTI-DT** has a quasi-one-relaxation channel. The population of the ICT and tightly bound excitonic states were found in the BDT-based copolymers, while only the latter was found in **PTI-DT**. The results confirm the importance of donor units in the synthesis of D–A copolymers for efficient ICT processes.

### 3.3. Thermal Properties of PBDTI-OT, PBDTI-DT and PTI-DT

**Figure 5:**
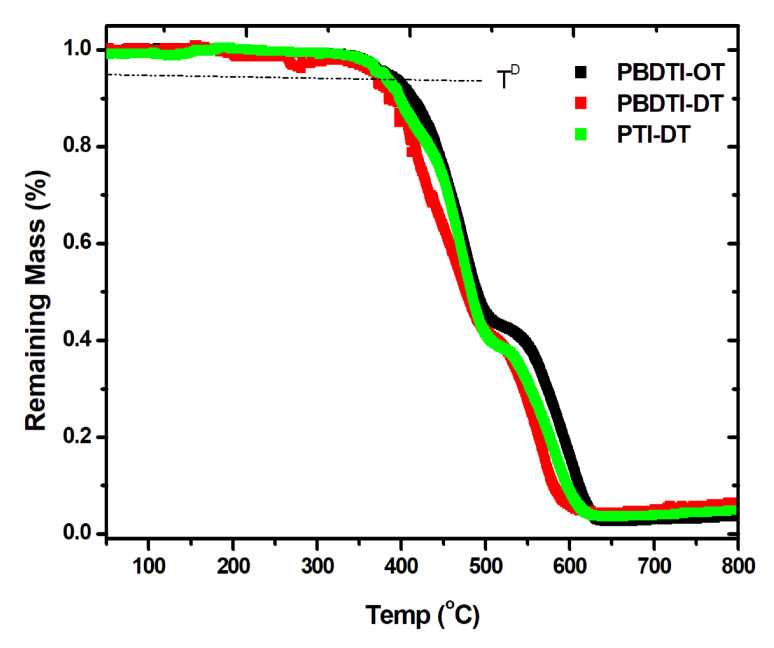
TGA measurement of **PBDTI-OT** (black), **PBDTI-DT** (red) and **PTI-DT** (green).

The thermal properties of **PBDTI-OT**, **PBDTI-DT** and **PTI-DT** were studied using TGA, as shown in Figure 5. The three copolymers have agood thermal stabilities, with decomposition temperatures ( T${}^{D}$ = 5% weight loss) above 380 °C. The thermal degradation in these materials follows two steps: one around 380 °C and the other above 550 °C. The first degradation accounts for the breaking up of the side chains. The shorter side chain in the isoindigo unit of **PBDTI-OT** slightly increased its thermal stability compared to **PBDTI-DT**, despite its lower molecular weight. The second thermal degradation is due to the decomposition of the backbone structures of the copolymers. The slight increase in the degradation temperature in **PBDTI-OT** also shows the strong bonding in its backbone structure compared to **PBDTI-DT** and **PTI-DT**. In general, the thermal stabilities of the copolymers make them applicable to flexible OSCs, which are normally processed at temeratures below 150 °C.

### 3.4. Structural Properties of PBDTI-OT, PBDTI-DT and PTI-DT Films

**Figure 6:**
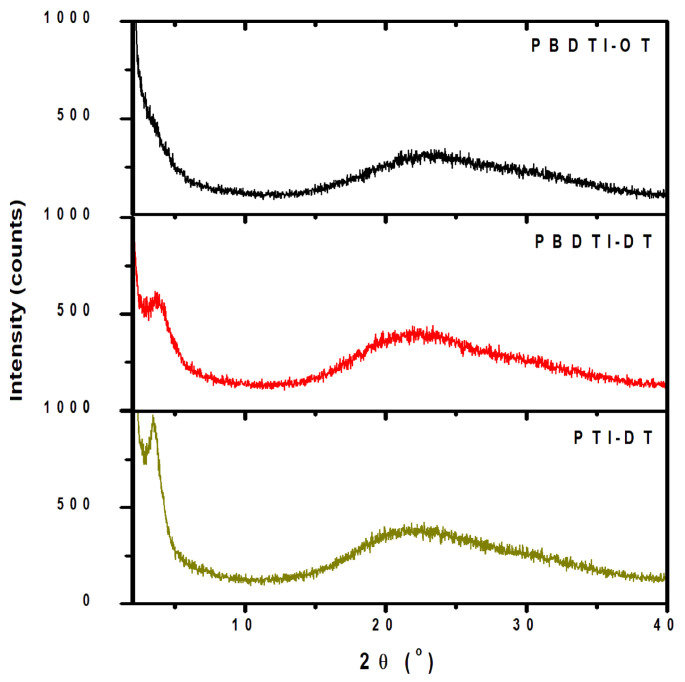
XRD traces **PBDTI-OT** (black), **PBDTI-DT** (red) and **PTI-DT** (dark yellow) in drop-casted films.

The structures of the copolymers were studied by powder XRD in drop-casted thin films, and the results are shown in Figure 6. The XRD traces of the three copolymers exhibit a broad peak (010) centered around 2θ = 22° due to the π−π stacking.

The calculated π−π stacking distances using Bragg’s equation Equation (1) are 0.38, 0.41 and 0.41 nm in **PBDTI-OT**, **PBDTI-DT** and **PTI-DT** films, respectively, confirming the presence of strong intermolecular interaction in the copolymer backbones [29]. **PBDT-DT** and **PTI-DT** exhibit a (100) diffraction peak at 2θ = 3.82° and 3.60°, corresponding to lammelar spacings of 2.3 and 2.5 nm, respectively. The inter-lammelar spacing in **PTI-DT** (2.5 nm) is higher than the well-studied thiophene-based homopolymer, poly-3-hexylthiophne (P3HT) (d = 1.63) [30]. This is due to the longer side chains both in the thiophene and isoindigo units of **PTI-DT** compared to P3HT. Note here that the (100) plane diffraction peak is sharper in **PTI-DT**, indicating its slightly higher crystallinity over the BDT-based copolymer. On the other hand, the BDT-based copolymer with a shorter side chain on the isoindigo unit (**PBDT-OT**) is fully amorphous. The higher crystallity of thiophene-based copolymer is beneficial for improved charge transport.

## 4. Conclusions

Three copolymers with BDT (**PBDTI-OD** and **PBDTI-DT**) and thiophene (**PTI-DT**) donor units and isoindigo acceptor units were designed and synthesized using the DAP method. The molecular weights of the BDT-based copolymers were more than two-fold higher than that of **PTI-DT**. The copolymers absorb in a wide range from from 300 to above 760 nm in thin films, making them low-band-gap polymers with E${}_{g}\approx$ 1.5 eV. The photophysical properties of the copolymers were studied in both films and solutions. Solvent polarity-dependent spectroscopic studies on **PBDTI-OD** and **PBDTI-DT** showed that the ground state is not sensitive to polarity of the environment, while the excited state is. This led us to conclude that the ground states of the BDT-based copolymers are non-polar. The PL spectra of the BDT-based copolymers were red-shifted with increasing solvent polarity of chlorinated solvents, confirming the dipolar characteristics of their first excited states. Therefore, the first excited state has ICT characteristics. However, the Stokes shift in **PBDTI-OD** and **PBDTI-DT** in the non-polar solvent, Chex, was not blue-shifted, as expected from its low polarity index. Self-aggregation of the copolymers in the poor solvent was found to favour the population of a tightly bound excitonic state, thereby inhibiting the generation of ICT state. The absorption and PL spectra of the thiophene-based copolymer were not influenced by solvent polarity. This led us to conclude that self-aggregation in the thiophene-based copolymer is induced despite the solvent polarity. Therefore, ICT state population in **PTI-DT** is not efficient. The copolymers showed excellent thermal stability, with a decomposition temperature above 380 °C. The XRD patterns of the three copolymers showed a halo from (010) plane due to π−π stacking. A second diffraction peak was observed in **PBDTI-DT** and **PTI-DT**, confirming a better crystallinity in these copolymers.

## Figures and Tables

**Table 1:** Molecular weight and yield data of **PBDTI-OD**, **PBDTI-DT** and **PTI-DT**.

Copolymer	Yield (%)	M${}_{n}$ (g/mol)	M${}_{w}$ (g/mol)	PDI
**PBDTI-OD**	56.25	16,932	53,461	3.2
**PBDTI-DT**	43.71	17,974	71,234	4.0
**PTI-DT**	66.07	12,537	23,574	1.9

**Table 2:** Electrochemical and Optical Properties of **PBDTI-OT**, **PBDTI-DT** and **PTI-DT**.

Name	$\lambda_{\max}$Soln (nm)		λ${}_{\max}$Film (nm)		^1^ Shift (nm)		${}^{2}$ $E_{g}$${}^{\mathrm{Opt}}$	$E_{\mathrm{HOMO}}$	$E_{\mathrm{LUMO}}$
	$\lambda_{\max1}$	$\lambda_{\max2}$	$\lambda_{\max1}$	$\lambda_{\max2}$	$\Delta \lambda_{\max1}$	$\Delta \lambda_{\max1}$	**eV**	**eV**	**eV**
**PBDTI-OD**	366, 440	631	374, 440	631	8, 0	0	1.53	−5.5	−3.9
**PBDTI-OT**	363, 437	628	370, 438	623	7, 4	−5	1.54	−5.7	−3.8
**PTI-DT**	—, 457	615	—, 461	623	-, 4	8	1.56	−5.6	−3.7

^1^ Abs peak shift from solution to film, ^2^ *E_g_* = 1200/*λ_onset_*.

## Data Availability

Data will be available based on request.
